# Imaging of Folate Receptor Expressing Macrophages in the Rat Groove Model of Osteoarthritis: Using a New DOTA-Folate Conjugate

**DOI:** 10.1177/1947603517738073

**Published:** 2017-11-03

**Authors:** Huub M. de Visser, Nicoline M. Korthagen, Cristina Müller, Ruud M. Ramakers, Gerard C. Krijger, Floris P. J. G. Lafeber, Freek J. Beekman, Simon C. Mastbergen, Harrie Weinans

**Affiliations:** 1 Department of Rheumatology and Clinical Immunology, University Medical Center Utrecht, Utrecht, The Netherlands; 2 Department of Orthopaedics, University Medical Center Utrecht, Utrecht, The Netherlands; 3 Department of Equine Sciences, Faculty of Veterinary Medicine, Utrecht University, Utrecht, The Netherlands; 4 Centre for Radiopharmaceutical Sciences ETH-PSI-USZ, Paul Scherrer Institute, Villigen-PSI, Switzerland; 5 MILabs B.V., Utrecht, The Netherlands; 6 Section of Radiation, Detection & Medical Imaging, Applied Sciences, Delft University of Technology, Delft, The Netherlands; 7 Department for Translational Neuroscience, Brain Center Rudolf Magnus, University Medical Center Utrecht, Utrecht, The Netherlands; 8 Department of Nuclear Medicine, University Medical Center Utrecht, Utrecht, The Netherlands; 9 Department of Biomechanical Engineering, Delft University of Technology, Delft, The Netherlands

**Keywords:** osteoarthritis, imaging, macrophages, inflammation, animal model

## Abstract

**Objective:**

To evaluate the presence and localization of folate receptor expressing macrophages in the rat groove model of osteoarthritis and determine the suitability of a new folate conjugate with albumin-binding entity (cm09) for *in vivo* SPECT (single-photon emission computed tomography) analysis.

**Design:**

In male Wistar rats, local cartilage damage was induced in addition to a standard (*n* = 10) or high-fat diet (*n* = 6). After 12 weeks, ^111^In labeled folate conjugates were administered, and SPECT/CT (computed tomography) imaging was performed after 24 hours. Subsequently, osteoarthritis severity and folate receptor expression were assessed using (immuno)-histological sections.

**Results:**

*In vivo* SPECT/CT imaging of the new folate conjugate (cm09) was as useful as a folate conjugate without albumin-binding entity in the groove model of osteoarthritis with less renal accumulation. Induction of cartilage damage on a standard diet resulted in no effect on the amount of folate receptor expressing macrophages compared with the contralateral sham operated joints. In contrast, inducing cartilage damage in the high-fat diet group resulted in 28.4% increase of folate receptor expression as compared with the nondamaged control joints. Folate receptor expressing cells were predominantly present in the synovial lining and in subchondral bone as confirmed by immunohistochemistry.

**Conclusions:**

Folate receptor expression, and thus macrophage activation, can clearly be demonstrated *in vivo*, in small animal models of osteoarthritis using the new ^111^In-folate conjugate with specific binding to the folate receptor. Increased macrophage activity only plays a role in the groove model of osteoarthritis when applied in a high-fat diet induced dysmetabolic condition, which is in line with the higher inflammatory state of that specific model.

## Introduction

The role of inflammation and inflammatory mediator production in osteoarthritis (OA) has been increasingly recognized.^1,2^ Inflammation of the synovial tissue is likely to contribute to the disease progression of OA and is implicated in many of the signs and symptoms of the disease, such as joint swelling and effusion.^3-5^ Synovial lining macrophages are considered to play an important role in the increased inflammation of the synovial tissue in OA, as these cells are known to produce pro-inflammatory cytokines (e.g., interleukin-1β and tumor necrosis factor-α) that diffuse into the cartilage through the synovial fluid.^2,5^ Moreover, synovial lining macrophages mediate osteophyte formation and fibrosis in early experimental OA.^6^ In cancer tissues the folate receptor-α is expressed, and on activated, but not resting, macrophages that are involved in inflammatory processes, the folate receptor-β is expressed.^7,8^

Folic acid has, even after conjugation to a therapeutic or diagnostic cargo, a high affinity for the folate receptor, and therefore, folic acid can be used as an targeting ligand for selective delivery of attached imaging and therapeutic agents to cancer tissues and sites of inflammation that contain activated macrophages.^9-13^ In rheumatoid arthritis (RA), an inflammatory joint condition where macrophages are thought to be the main promoter of disease activity, folate receptor expression on activated macrophages can be visualized using radiolabeled folate.^14,15^ Recently, activated macrophage involvement was observed with SPECT/CT (single-photon emission computed tomography) imaging with Etarfolatide in symptomatic OA patients.^16^ Besides, SPECT/CT imaging can also be used in experimental OA, where *in vivo* imaging and quantification of macrophage activity is feasible.^17,18^ However, the increase in macrophage activity in OA is modest compared with RA, and the specific role of the folate receptor expressing macrophages in the disease process of OA is not yet elucidated. A limitation of folate-based nuclear imaging is the fact that in its (clinical as well as preclinical) application, folate radioconjugates accumulate in the renal tissue.^19^ This is mostly a consequence of the rapid clearance of folic acid conjugates from the blood circulation and specific binding to the folate receptor, which is expressed in the renal proximal tubular cells.^19^ Therefore, a novel DOTA-folate conjugate (cm09) with a low-molecular-weight albumin-binding entity was recently developed. This additional functionality was shown to increase the distribution of folate radioconjugates in the target tissue while retention of activity in the kidneys was reduced.^20^ In particular, when the amount of macrophages may be low in the joint, such as in OA, this new radioconjugate has potential for improved imaging of the diseased site. The rat groove model, where cartilage damage is mechanically induced on the femoral condyles with minimal joint inflammation and mild joint degeneration, is a model better resembling the human low-inflammatory OA situation of slow-progressive disease development.^21^ On the other hand, when cartilage damage is mechanically induced in addition to high-fat (HF) diet feeding, an inflammatory driven increase in joint degeneration was observed.^22^ The role of folate receptor expressing macrophages in both OA models is currently unknown. We hypothesized that accumulation of the novel folate radioconjugate in the target tissue may be visualized in the rat groove model on both a standard diet and a HF diet.

Therefore, the aim of this study was to determine the presence and specific localization of folate receptor expressing macrophages in the rat groove model of OA in combination to a standard or HF diet. Additionally, we evaluated the *in vivo* characteristics of the new ^111^In-folate-conjugate (^111^In-cm09) comprising an albumin-binding entity in experimental early-OA compared with a conventional folate radioconjugate compound without albumin-binding entity in the standard diet fed rats.

## Methods

### Study Design

**Figure 1.**
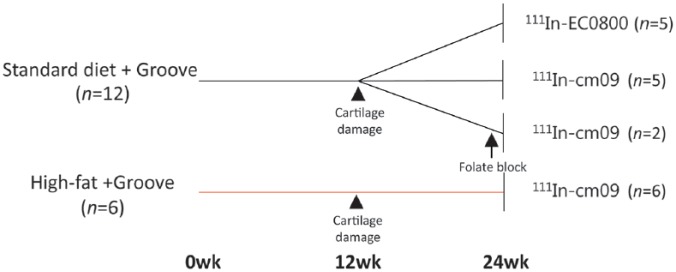
Experimental design of the study. OA was induced by mechanically induced cartilage damage according to the groove model in one knee joint with sham surgery on the contralateral knee joint (*n* = 12). The folate conjugate with albumin-binding entity (cm09) and the conventional folate conjugate (EC0800) were labeled with ^111^In and subsequently injected intravenously 12 weeks after the induced cartilage damage. In 2 rats an excessive amount of folic acid was administrated prior to injection of the folate conjugate to check its specificity by blocking the receptor. In parallel, cartilage damage was induced in addition to a high-fat diet (*n* = 6). Twenty-four hours after intravenous injection of both folate radioconjugates a SPECT/CT scan was performed.

In eighteen, 24-week-old, male Wistar rats (Charles River, Sulzfeld, Germany), cartilage damage was mechanically induced on the femoral condyle in one knee joint, according to the groove model as previously described.^21^ Sham surgery was performed on the contralateral knee joints as an internal control. In addition, 6 rats were fed a HF diet (D12492i, Research Diets Inc., New Brunswick, NJ) after randomization, 12 weeks prior to the mechanically induced cartilage damage, and the HF diet was continued for 12 weeks after induction of cartilage damage. In the HF diet group solely the new ^111^In-folate-conjugate (^111^In-cm09) comprising an albumin-binding entity was used to look at the macrophage activity in this model. A detailed overview of the study setup is given in **Figure 1**. All animals had access to food pellets and tap water *ad libitum* and were housed 2 per cage. All procedures were approved by the Utrecht University Medical Ethical Committee for Animal Studies (DEC 2014.I.03.019).

### Radiosynthesis

Stock solutions of DOTA-folates (EC0800 provided by Endocyte Inc., USA, and cm09 provided by Dr. C. Müller, Center for Radiopharmaceutical Sciences ETH-PSI-USZ, Switzerland) were mixed with sodium acetate (0.5 M, pH 8) and 60 MBq of ^111^InCl_3_ (Mallinckrodt-Tyco, Petten, the Netherlands), resulting in a specific activity of 10 MBq/nmol for EC0800 and 20 MBq/nmol for cm09. The reaction mixture was incubated at 90°C for 10 minutes. Labeling quality control was performed by high-performance liquid chromatography (HPLC; Dionex UltiMate 3000, Thermo Scientific) connected to a gamma detector using an Acclaim 120 stainless steel C18 column (120 A, 3 µm, 4.6 × 150 mm). Elution (1 mL/min) was performed using 0.1% TFA H_2_O (A) and acetronitrile (B) as eluents and a gradient from 95% A and 5% B to 20% A and 80% B in 20 minutes at 25°C. Radiolabeling of compound cm09 resulted in a higher radiochemical purity as compared with ^111^In-EC0800 as determined by HPLC (98% vs. 86%). Subsequently, a solution of 10 µL sodium-diethylenetriaminepentaacetic acid (Na-DTPA 5 mM) was added for complexation of remaining traces of free ^111^In. Finally, the solution was further diluted in 600 µL of saline for injection (46-67 MBq injected activity).

### SPECT/CT Imaging and Data Analysis

Twelve weeks after groove surgery to induce OA, all animals were randomized, sedated, and the radiolabeled compounds were injected in the lateral tail vein 24 hours prior to SPECT/CT for both folate radioconjugates. SPECT/CT imaging was performed using a dedicated small animal PET/SPECT/CT scanner (VECTor^4^CT scanner, MILabs B.V., Utrecht, The Netherlands) using 173 keV ± 10% and 247 keV ± 10% energy windows for ^111^In. Two adjacent background windows per photo peak were used for triple energy window scatter and cross-talk correction. The system was fitted with a general purpose pinhole collimator (GP-RM) with 75 pinholes of 1.5-mm diameter suited for both sub-mm total body and sub-mm focused knee imaging. To maximize image quality at low isotope concentrations, we choose to use a relative long acquisition time of 52 minutes (45 minutes SPECT and 7 minutes µCT imaging) per total body scan and 49 minutes (45 minutes SPECT and 4 minutes µCT imaging) for the focused knee scans. SPECT images were reconstructed using iterative Maximum Likelihood Expectation Maximization reconstructions with resolution recovery (MILabs Rec 7.00 software^23^) at an isotropic 0.8-mm voxel grid width and 100 iterations. Reconstructed volumes of SPECT scans were postfiltered with an isotropic 3-dimensional Gaussian filter of 1 mm full width of half maximum. For 3D visual representation Osirix software (Osirix 8.0.2) was used. Corresponding CT scans were acquired at 55 kV and 0.19 mA. To calculate the uptake of radiolabeled folate, the registered CT and quantified SPECT images were analyzed using PMOD biomedical image analyzing software (PMOD 3.8, Zurich, Switzerland). An ellipse-shaped volume of interest (VOI) was drawn, on CT data, unaware of the activity on the SPECT, as previously described.^17,24^ To reduce interindividual variation, all data are presented as percentage of the injected activity corrected per selected tissue volume (%IA/cm^3^). In addition, the specificity of the technique was tested by blockade of the folate receptor by injection of a 500-fold excess of folic acid (100 µg in 100 µL phosphate-buffered saline) immediately before radiofolate administration in 2 animals with mechanically induced cartilage damage on a standard diet.

### Histopathological and Immunohistochemical Examination of the Knee Joint

Immediately after SPECT/CT imaging, both knee joints were processed for histological evaluation according to the guidelines of the OARSI histopathology score for rats.^25^ Lateral plane sections of 5 µm thickness were made. For histopathological examination, the slides were stained with both hematoxylin-eosin for synovial inflammation and safranin-O with a fast-green counterstain to envision the amount and distribution of the glycosaminoglycan. Joint degeneration was evaluated using the rat OARSI histopathology score.^25^ The surgical applied grooves were not taken into account as part of the histological score, but only the direct adjacent articular cartilage was evaluated. CD68 and folate receptor were stained by immunohistochemistry. All sections were blocked for nonspecific binding with endogenous enzyme block (Dako S2003, Glostrup, Denmark) following antigen retrieval. CD68 was retrieved by incubation with pepsin 0.1% at 37° for 30 minutes and folate receptor was retrieved by 0.01 M citrate buffer, pH 6.0. Sections were incubated 1:250 with primary antibodies for CD68 (ab31630, Abcam, Cambridge, UK) and 1:200 with folate receptor (orb156919, Biorbyt, Cambridge, UK) all at 4°C overnight. Subsequently, the antibody was visualized with Envision HRP anti-mouse (Dako) for CD68 and Envision HRP anti-rabbit (Dako) for folate receptor 30 minutes at room temperature following a 5-minute conversion of DAB. Sections were counterstained with Mayer’s hematoxylin and isotype control stainings were carried out.

### Statistical Analysis

Histological data are presented as mean values with 95% confidence interval of the mean for both knee joints. All quantified data of the SPECT/CT images were reported as percentage of the injected dose per cm³ (%IA/cm^3^) with 95% confidence interval of the mean. Comparison between experimental and contralateral control knee joints for each folate conjugate in the groove model was performed by the paired samples *t* test. The independent samples *t* test was used to compare between both folate conjugates and between the standard and HF diet rats (SPSS Statistics 21, SPSS Inc., Chicago, IL).

## Results

### Joint Degeneration

In the group with mechanically induced cartilage damage by groove surgery on a standard diet, a mild increase in joint degeneration was observed compared with the sham operated contralateral control knee joints (3.8 [2.4-4.5] vs. 1.6 [0.4-2.7]; *P* = 0.026). Besides, no increase in synovial membrane inflammation was observed in the experimental knee joints compared with the sham operated knee joints (0.3 [−0.1 to 0.6] vs. 0.1 [−0.1 to 0.3]; *P* = 0.32). When mechanically induced cartilage damage was performed in addition to a HF diet a more pronounced increase in joint degeneration was observed 12 weeks postsurgery, compared with the sham operated contralateral controls (6.8 [4.8-8.8] vs. 2.0 [0.8-3.2]; *P* = 0.011), together with an increase in synovial membrane inflammation compared with the contralateral sham operated knee joints (2.2 [1.7-3.1] vs. 0.8 [0.2-1.4]; *P* = 0.025).

### Macrophage Activity

**Figure 2.**
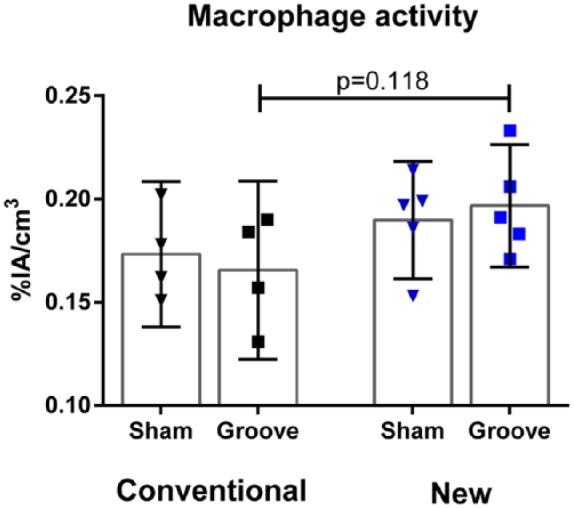
Observed macrophage activity between the new folate radioconjugate with albumin-binding entity (cm09) and the conventional folate radioconjugate (EC0800) for the knee joints with mechanically induced cartilage damage by groove surgery on a standard diet. The data are presented for the 2 different folate conjugates and the bars represent mean activity with 95% confidence interval of the mean. Differences in macrophage activity between the experimental grooved knee joints compared with the sham controls were determined by the paired samples *t* test. The difference between the new folate conjugate with albumin-binding entity compared with the conventional folate conjugate without albumin-binding entity was determined by the independent samples *t* test.

**Figure 3.**
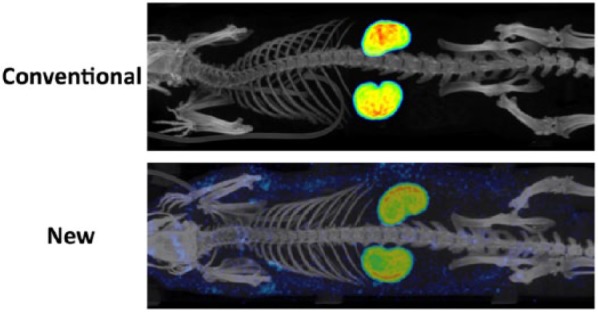
Rat total body SPECT/CT reconstructions, 12 weeks after mechanically induced cartilage damage was performed in addition to a standard diet. The SPECT/CT was performed 24 hours after intravenous administration of the conventional folate radioconjugate EC0800 (**above**) and new folate radioconjugate cm09 with albumin-binding entity (**below**). Images show a reduced activity in the kidneys of the rat injected with the folate radioconjugate with albumin-binding entity. Besides, a higher amount of activity in the blood circulation and subcutaneous can be appreciated from these images when the new folate conjugate was applied.

Each animal received 53 ± 9 MBq of either radioconjugates, injected intravenous in the lateral tail vein. Looking specifically at the effect of the new folate conjugate with albumin-binding entity compared with the conventional folate conjugate, no difference in macrophage activity was detected in the mechanically induced groove model on a standard diet (0.197%IA/cm^3^ [0.167-0.227] vs. 0.165 [0.122-0.209]; *P* = 0.118; **Fig. 2**). However, despite no effect in macrophage activity, the renal accumulation was decreased by 60% for ^111^In-cm09 comprising an albumin-binding entity as compared with the conventional radiofolate, 24 hours after intravenous administration (3.3 and 3.0%IA/cm^3^ vs. 8.1 and 8.1%IA/cm^3^; **Fig. 3**). Addition of an excessive amount of unlabeled folic acid resulted in an activity in the knee joints of 0.09%IA/cm^3^ (0.07-0.10) and was not different between the experimental and sham operated control knee joints (*P* = 0.456). This clearly confirms specific binding of the radiofolates to the folate receptor.

**Figure 4.**
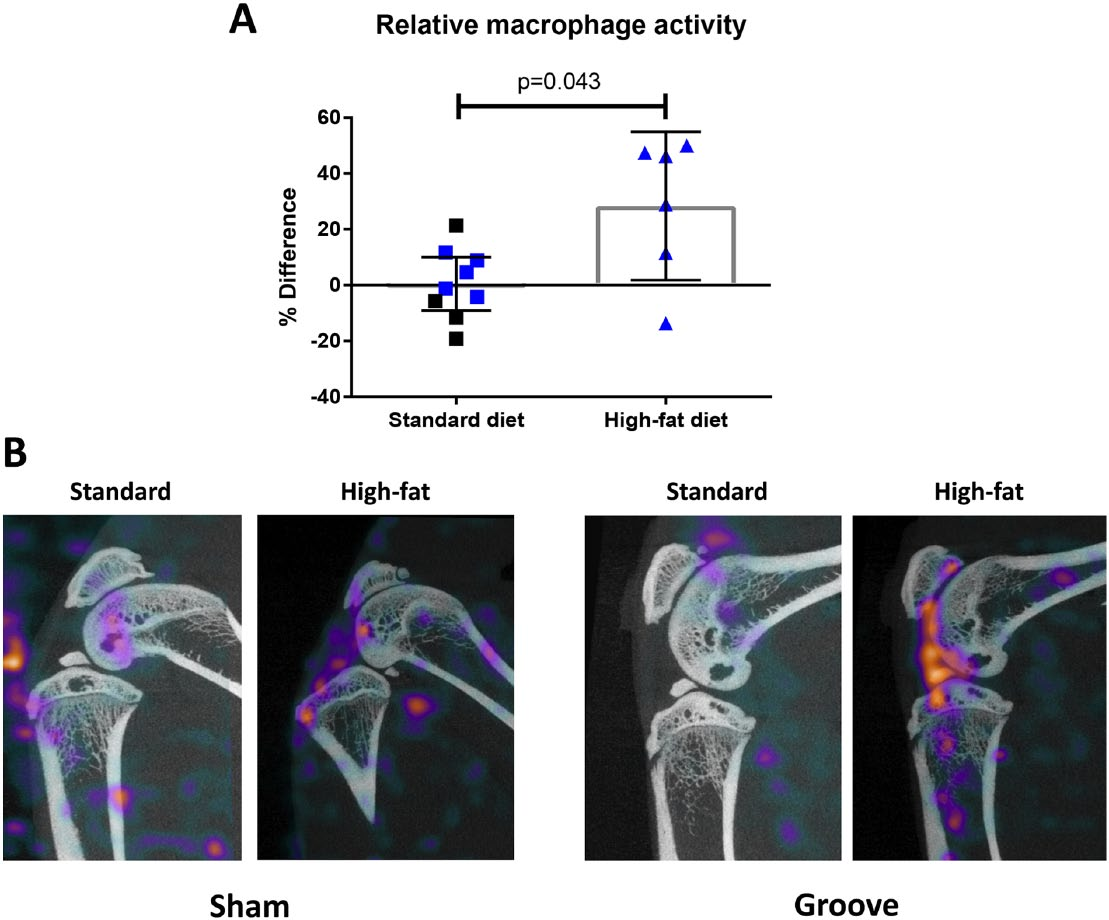
Relative difference in macrophage activity between experimental knee joints with mechanically induced cartilage damage and sham operated controls for each animal in both conditions (**A**). The bars represent mean change with 95% confidence interval of the mean. Blue data points indicate rats with the new folate conjugate with albumin-binding entity, whereas the black data points represent the conventional folate conjugate without albumin-binding entity. *P* value indicates a statistically significant difference as determined by the independent samples *t* test. Representative SPECT/CT reconstructions of sham operated control knee joints (**left**) and the mechanically induced cartilage damage (**right**), 24 hours after injection of folate radioconjugates for both conditions (**B**). Here, the single slice CT through the center of the joint is visualized with the sum of the activity over the corresponding SPECT slices showing macrophage activity of the entire joint. When groove surgery was combined with a HF diet most activity is seen in the knee joint, whereas the other joints have subcutaneous activity as well.

When the macrophage activity in all knee joints with induced cartilage damage on a standard diet was compared with the sham operated contralateral controls, no differences were observed (+0.48%%IA/cm^3^ [−9.10 to 10.07]; *P* = 0.949; **Fig. 4**). However, when mechanically induced cartilage damage was performed in combination to a HF diet, increased macrophage activity was observed compared with the sham operated control knee joints (+28.37%%ID/cm^3^ [1.81 to 54.93]; *P* = 0.058; **Fig. 4A** and **B**). Moreover, when the relative folate receptor expressing macrophage activity in the HF diet rats was compared with standard diet fed rats, a statistical significant increase was observed (*P* = 0.043; **Fig. 4**).

### Immunohistochemistry

**Figure 5.**
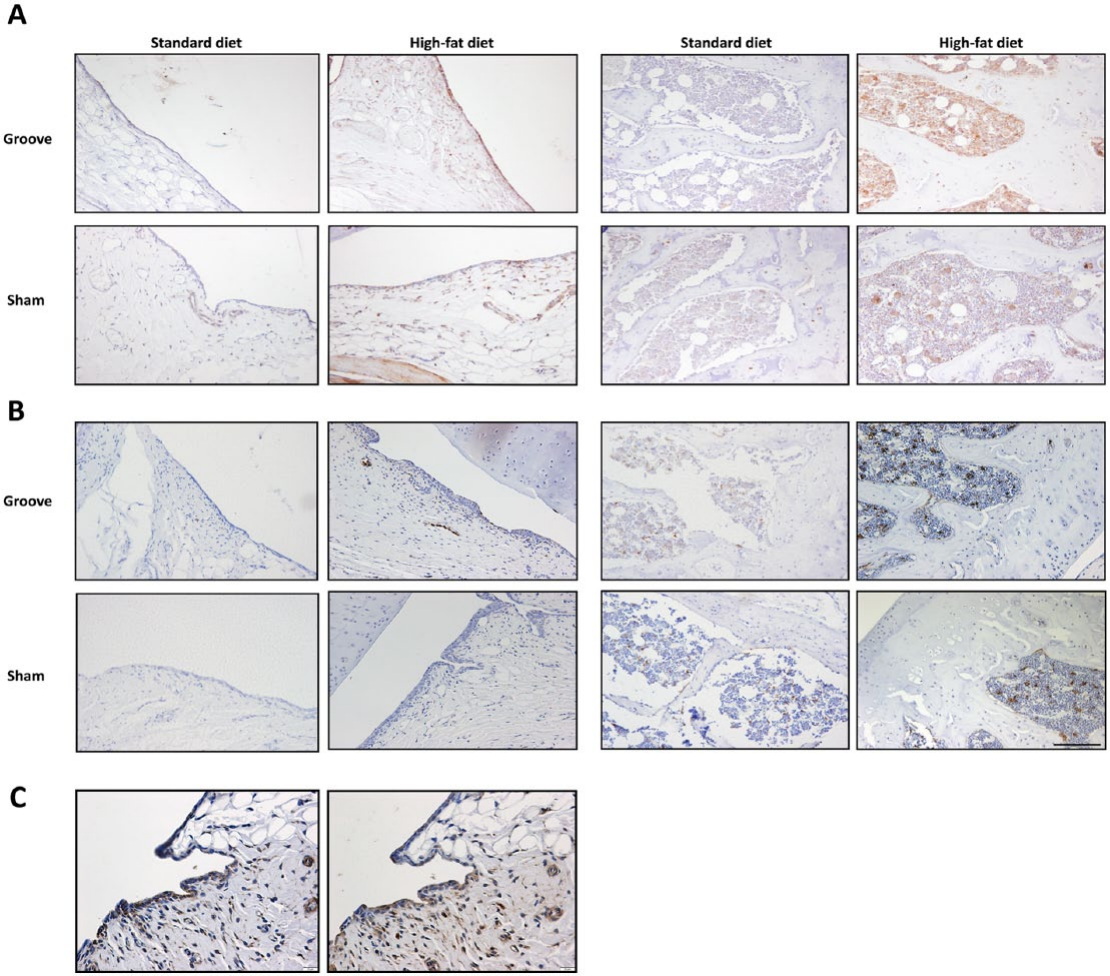
Expression of folate receptor (**A**) and CD68 (**B**) by immunohistochemical analysis on paraffin-embedded slides from rat knee joints. Representative immunostaining images originating from the synovial membrane as well as the subchondral bone of the tibia are shown for the experimental grooved and contralateral sham control knee joints for both types of diet. Brown staining indicates positive cells and are highlighted by the arrows. And the localization of both Folate receptor (**left**) and CD68 (**right**) antibodies in sequential tissue sections is presented (**C**). Scale bar is 100 µm (**A** and **B**) and 20 µm (**C**).

Immunostains revealed only minor expression of folate receptor in the synovial membrane in knee joints with mechanically induced cartilage damage as well as in the sham operated contralateral control joints for the rats that were fed a standard diet (**Fig. 5A**). When mechanically induced cartilage damage was performed in combination to a HF diet, an increased expression of the folate receptor was observed in the inflamed synovial lining. Besides, increased activity was observed in the subchondral bone in the HF diet fed rats with a further increased activity in the experimental grooved knee joints compared with the sham operated controls (**Fig. 5A**). The additional CD68 staining confirmed the co-localization of CD68-positive cells on similar locations as observed in the folate receptor staining of the synovial membrane as well as the subchondral bone, suggesting the folate receptor-positive cells were mainly activated macrophages (**Fig. 5B**). There was an increased expression of CD68 in the HF diet compared with the standard diet fed rats, especially in the subchondral bone.

## Discussion

Folate receptor expressing macrophages could be observed in the rat groove model of OA, a mechanical driven noninflammatory model, but did not result in an increased accumulation of radiofolates compared with the sham operated controls. Groove surgery in addition to HF diet feeding, a model driven by local inflammatory changes, did, however, result in increased macrophage expression as shown by increased radiofolate accumulation visualized by SPECT/CT and further corroborated by increased signal in immunohistochemistry samples. Macrophage activation can clearly be demonstrated *in vivo*, using the new ^111^In-folate conjugate (^111^In-cm09) comprising an albumin-binding entity in small animal OA models as well as with conventional folate conjugate.

Currently there are no validated (longitudinal) measures of inflammatory activity in the synovium of OA joints available.^26^ However, recently a direct *in vivo* indication of activated macrophage involvement in human OA was presented.^16^ This suggests the possibility of macrophage-targeted monitoring and therapy for a subgroup of OA patients, with inflammation related to macrophages, who are at risk for disease progression.^16^ However, as the inflammatory state of OA joints is considered mild,^27,28^ the number of activated macrophages in the joints are limited compared with other conditions (e.g., RA and cancer-associated macrophages).^4^ Therefore, it is assumed that macrophage visualization in OA patients will be challenging. Experimental models of OA can be of additional value in this perspective. SPECT/CT imaging has previously demonstrated the role of activated macrophages in murine and rat models of biochemical and posttraumatic OA.^17,29^ This is the first time the surgical rat groove model, better resembling the human noninflammatory OA situation of slow-progressive disease development, was used to study macrophage activity. Especially in this mild inflammatory condition, it was anticipated that the new folate conjugate with albumin-binding entity could potentially be more sensitive to detect activated macrophages compared with the use of a conventional folate radioconjugate. Nevertheless, no difference in the signal of accumulated radiofolate was observed when local cartilage damage was induced in addition to a standard diet compared with the control joints with sham surgery. A possible explanation for this observation is the negligible inflammatory state of these joints that was comparable to the controls, in line with earlier studies using this model.^21,30^ Due to the serum protein-binding characteristics of the new folate conjugate, the kidney accumulation was also significantly reduced in this preclinical OA model compared with the radiofolate without albumin-binding entity. As such, this is an important advantage of the new folate conjugate, as radiation exposure is an important limitation of monitoring folate receptor expressing macrophages using SPECT/CT, because of the known accumulation in the renal tissue of folate based radiopharmaceuticals.^31^

Studying the activated macrophage involvement in the disease process of OA remains challenging as the amount of inflammation is limited and the exact involvement in the process of OA is currently unclear. The moment macrophages are recruited into cytokine expressing tissues they become activated.^32^ Their numbers increase massively in inflammation and have the capacity to influence tissue remodeling and facilitate repair in sites of injury.^4^

To better study the role of macrophage activation in the disease process of OA, we therefore included the model where mechanically induced cartilage damage is performed in addition to a HF diet. We previously showed that the observed progression of joint degeneration in this model was mainly inflammatory driven.^22^ We observed a clear increase of activated macrophages in the specific joint tissues compared with sham operated contralateral controls.

In the disease process of OA, activated macrophages are predominantly present in the synovial lining layer and express the folate receptor, which binds folate conjugates.^33^ In the present study, we confirmed by immunostaining that folate receptor-positive macrophages are mainly present in the synovial lining layer as seen by SPECT/CT, in an inflammatory driven experimental model of OA and consistent with expression of CD68 as previously reported.^34,35^ Interestingly, we observed not only increased folate receptor expression in the synovial lining but also in the bone marrow of the subchondral bone with co-expression of CD-68 in the HF diet fed rats. Although the folate receptor was not detected on osteoclasts,^36^ mononuclear osteoclast precursors could explain this increased expression. Mononuclear osteoclast precursors in OA synovial tissue express the macrophage marker CD14, which is known to co-express the folate receptor.^37,38^

The determination of the exact location of activated macrophages expressing the folate receptor is an essential first step in identifying the role of these macrophages in the disease process of OA. Next, the focus should be on identifying the specific macrophages that express the folate receptor and elucidating their phenotype.

Previously, activated macrophages have been grouped to their response to either pro-inflammatory (M1) or anti-inflammatory (M2) characteristics.^38,39^ Recent findings show that the folate receptor is especially upregulated by M2 macrophages.^29,34,38,40^ The anti-inflammatory aspects of the folate receptor-positive M2 macrophages suggest that their presence is potentially beneficial to slow down the disease process as determined by the pro-inflammatory environment of the joint. However, a more specific distinction of macrophage subsets expressing the folate receptor is necessary to develop possible targeted therapies in the future.

The present study showed that the albumin-binding folate radiotracer ^111^In-cm09 is suitable for SPECT imaging in small animal models of OA. Besides, the new folate conjugate is safe to use and well tolerated in these animals in addition to reduced renal accumulation of radioactivity as compared with conventional radiofolates. In the groove model, where cartilage damage is mechanically induced, in addition to a standard diet, no difference in macrophage activity was observed. However, HF diet feeding in combination with local cartilage damage resulted in an increased macrophage activity compared with standard diet fed rats. In addition, immunostaining suggests the folate receptor-positive cells observed in the knee joints are activated macrophages mainly present in the synovial lining. Taken together, our findings indicate that the new folate radioconjugate is a useful tool to study the role of activated macrophages in the disease processes of OA.
